# Regional differences in mitochondrial DNA methylation in human post-mortem brain tissue

**DOI:** 10.1186/s13148-017-0337-3

**Published:** 2017-05-03

**Authors:** Matthew Devall, Rebecca G. Smith, Aaron Jeffries, Eilis Hannon, Matthew N. Davies, Leonard Schalkwyk, Jonathan Mill, Michael Weedon, Katie Lunnon

**Affiliations:** 1 University of Exeter Medical School, RILD, University of Exeter, Barrack Road, Devon, UK; 2 Institute of Psychiatry, Psychology and Neuroscience, King’s College London, De Crespigny Park, London, UK; 3 Department of Twin Research & Genetic Epidemiology, King’s College London, Lambeth Palace Road, London, UK; 4 School of Biological Sciences, University of Essex, Essex, UK

**Keywords:** 5-mC, 5-Methylcytosine, Blood, Brain, DNA methylation, Epigenetics, MeDIP-seq, Mitochondria, *NUMT*s

## Abstract

**Background:**

DNA methylation is an important epigenetic mechanism involved in gene regulation, with alterations in DNA methylation in the nuclear genome being linked to numerous complex diseases. Mitochondrial DNA methylation is a phenomenon that is receiving ever-increasing interest, particularly in diseases characterized by mitochondrial dysfunction; however, most studies have been limited to the investigation of specific target regions. Analyses spanning the entire mitochondrial genome have been limited, potentially due to the amount of input DNA required. Further, mitochondrial genetic studies have been previously confounded by nuclear-mitochondrial pseudogenes. Methylated DNA Immunoprecipitation Sequencing is a technique widely used to profile DNA methylation across the nuclear genome; however, reads mapped to mitochondrial DNA are often discarded. Here, we have developed an approach to control for nuclear-mitochondrial pseudogenes within Methylated DNA Immunoprecipitation Sequencing data. We highlight the utility of this approach in identifying differences in mitochondrial DNA methylation across regions of the human brain and pre-mortem blood.

**Results:**

We were able to correlate mitochondrial DNA methylation patterns between the cortex, cerebellum and blood. We identified 74 nominally significant differentially methylated regions (*p* < 0.05) in the mitochondrial genome, between anatomically separate cortical regions and the cerebellum in matched samples (*N* = 3 matched donors). Further analysis identified eight significant differentially methylated regions between the total cortex and cerebellum after correcting for multiple testing. Using unsupervised hierarchical clustering analysis of the mitochondrial DNA methylome, we were able to identify tissue-specific patterns of mitochondrial DNA methylation between blood, cerebellum and cortex.

**Conclusions:**

Our study represents a comprehensive analysis of the mitochondrial methylome using pre-existing Methylated DNA Immunoprecipitation Sequencing data to identify brain region-specific patterns of mitochondrial DNA methylation.

## Introduction

Mitochondria are unique organelles in that they have their own circular genome, approximately 16.6 kb in size [1]. Mitochondrial DNA (mtDNA) consists of 37 genes, 22 encoding for transfer RNAs (tRNAs), two for ribosomal RNAs (rRNAs) and 13 encoding for proteins important in the electron transport chain. Each of these 13 proteins are directly involved in the regulation of cellular respiration, generating the majority of ATP required for the process. However, mitochondria have an array of other important cellular roles such as calcium homeostasis [2] and neural stem cell differentiation [3]. As such, abnormal mitochondrial function, dynamics and trafficking have been associated with a number of brain disorders including Alzheimer’s disease [4, 5], schizophrenia [6], bipolar disorder [7] and major depressive disorder [8].

Epigenetic processes mediate the reversible regulation of gene expression, occurring independently of DNA sequence variation, acting principally through chemical modifications to DNA and nucleosomal histone proteins and orchestrate a diverse range of important physiological functions. DNA methylation is the best characterized and most stable epigenetic modification modulating the transcription of mammalian genomes and, because it can be robustly assessed using existing genomic DNA resources, is the focus of most human epidemiological epigenetic research to date [9]. The most widely used method for epigenome-wide analysis of DNA methylation is the Illumina 450K methylation array, and a number of studies have recently shown differential DNA methylation of the nuclear genome (ncDNA), between different tissue types [10–12] and also in a range of complex diseases, from brain disorders such as Alzheimer’s disease [13–15] and schizophrenia [16, 17], to systemic diseases such as type 2 diabetes [18] and Crohn’s disease [19]. However, with no representation of the mitochondrial genome on this platform, as well as a lack of analysis on other genome-wide platforms, the role of mtDNA methylation has been largely neglected [20, 21].

Since the identification of 5-methylcytosine (5-mC) in mitochondria, research into mtDNA methylation as an independent and potentially relevant mark has received more regular attention [22, 23]. However, most research is either focussed on low resolution, global DNA methylation, or candidate gene DNA methylation changes using techniques such as bisulfite pyrosequencing [20]. These recent publications have indicated that differences in mtDNA methylation are present in a variety of different phenotypes [24–29] and may have potential utility as a biomarker [30]. In addition, a recent study has explored the use of Methylated DNA Immunoprecipitation Sequencing (MeDIP-seq) to investigate changes in mtDNA methylation across 39 cell lines and tissues from publicly available data [31]. At present, genome-wide sequencing technologies have not yet been used to interrogate alterations in the mtDNA methylome across tissues in the same individuals.

A high proportion of current, publicly available, genome-wide DNA methylation data has been generated through the use of MeDIP-seq, a method designed to interrogate genome-wide changes in methylation at high throughput and low cost [32]. However, given the presence of nuclear-mitochondrial pseudogenes (*NUMT*s), regions of the nuclear genome that share a high sequence homology with their mitochondrial paralogue [33, 34], mitochondrial reads are often discarded from further analysis. The development of bioinformatic pipelines to investigate regions of differential mtDNA methylation from whole genome data would provide a novel way in which to interrogate the mtDNA methylome in publicly available data. Here, we control for the presence of *NUMT*s in a previously published MeDIP-seq dataset, to investigate differential DNA methylation across the mitochondrial genome in human post-mortem brain samples.

## Results

### MtDNA methylation patterns are correlated between the cortex, cerebellum and blood

**Fig. 1:**
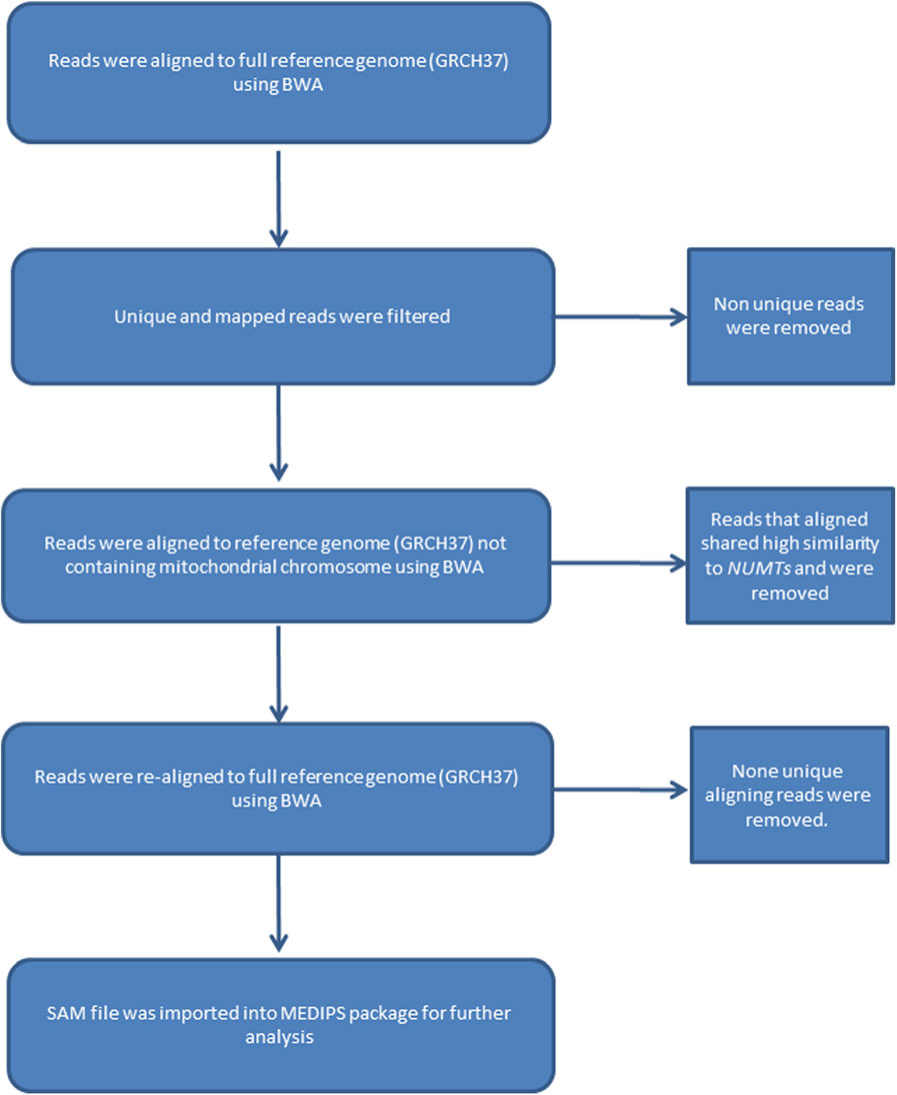
Overview of the analysis pipeline

**Fig. 2:**
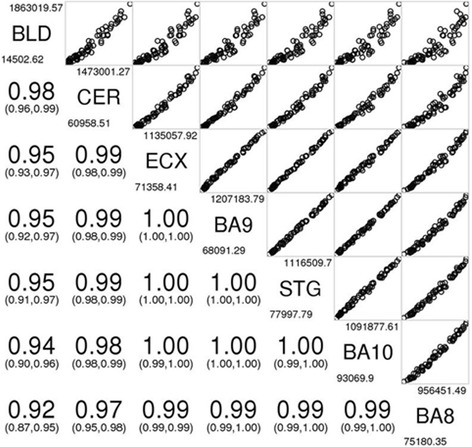
MtDNA methylation patterns are correlated between the cortex, cerebellum and blood. Samples were ordered based upon the similarity of their principal components, RPKM values, with *r* calculated for the correlations between each tissue. *BLD* blood, *BA8* Brodmann area 8, *BA9* Brodmann area 9, *BA10* Brodmann area 10, *CER* cerebellum, *CTX* cortex, *ECX* entorhinal cortex, *STG* superior temporal gyrus

To date, no study has investigated differences in mtDNA methylation across different matched regions of human brain and blood samples. Our sample (Table 1) consisted of MeDIP-seq data from three individuals, free of any neuropathology and neuropsychiatric disease, for five different regions of the cortex (Brodmann areas (BA) 8, 9 and 10, superior temporal gyrus (STG) and entorhinal cortex (ECX)), the cerebellum (CER) and pre-mortem blood [35]. Given that MeDIP-seq data has been generated from standardly extracted total genomic DNA and thus contains a mixture of ncDNA and mtDNA [36], we initially controlled for regions of high sequence homology between the two genomes within our data by realigning mtDNA reads to a series of custom reference genomes using an in-house pipeline (see the Methods section) to specifically analyze mtDNA methylation (Fig. 1). Briefly, after an initial alignment to the GRCH37 reference genome using BWA, uniquely mapped reads were extracted and aligned to a custom GRCH37 reference genome not containing the mitochondrial sequence. Reads that did not map to this custom genome were found to share less homology with the nuclear genome and were taken forward and realigned once more to the full reference genome. Initially, we were interested to investigate whether changes in mtDNA across the mitochondrial genome were highly correlated between different tissue types. Using principal component analysis (PCA), we found that mtDNA methylation patterns are highly correlated between different cortical regions (*r* > 0.99, *p* < 2.2E−16), with a slightly weaker correlation between the cerebellum and cortex (*r* > 0.97, *p* < 2.2E−16) (Fig. 2). Due to the small number of blood samples available, deriving a significance level for the correlations between the cerebellum and blood could not be made. Instead, in an attempt to explore the similarity between matched blood and cerebellum samples, the direction of differential methylation with respect to the cortex was used. Here, we found that 93.1% of the windows analyzed in the cerebellum and blood had the same direction of methylation difference with respect to the cortex, further suggesting a strong correlation between the two tissue types.

**Table 1:** Demographic information

Individual	Age at death (years)	Age at bloods sampled (years)	Post-mortem delay (hours)	Gender
1	82	79	43	Female
2	92	N/A	17	Female
3	78	78	10	Male

MeDIP-seq data was available from post-mortem brain samples obtained from three individuals free of any neuropathology and neuropsychiatric disease. Data was available for five different regions of the cortex (Brodmann areas (BA) 8, 9 and 10, superior temporal gyrus (STG), entorhinal cortex (ECX), the cerebellum (CER) and pre-mortem blood (BLD). MeDIP-seq data was available for all individuals from cortical and cerebellar samples; however, blood MeDIP-seq data was not available for individual 2. Data is freely available to download from http://epigenetics.iop.kcl.ac.uk/brain

### Differentially methylated regions of the mitochondrial genome can be identified between anatomically distinct cortical regions and the cerebellum

**Fig. 3:**
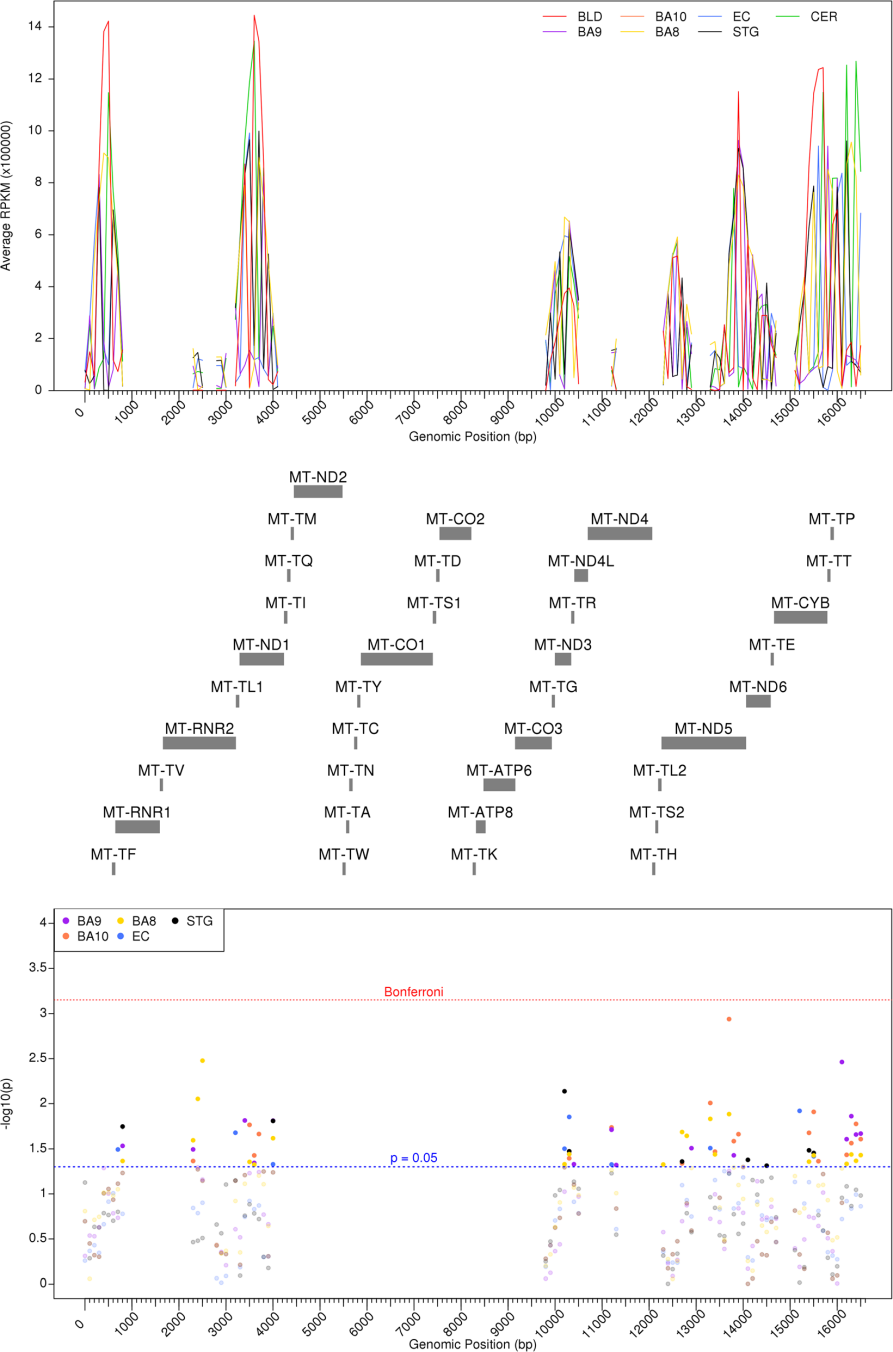
DNA methylation differences are seen in the mitochondrial genome between brain regions and blood. Average raw RPKM values across the mitochondrial genome for each individual cortical brain region alongside matched blood and cerebellum samples are shown in the *top panel*, with gene positions downloaded from GENCODE shown in the *middle panel*. For each 100 bp window, paired *t* tests were performed to compare each cortical brain region and the cerebellum, with -log10 (*p*) shown in the *bottom panel*. *BLD* blood, *BA8* Brodmann area 8, *BA9* Brodmann area 9, *BA10* Brodmann area 10, *CER* cerebellum, *CTX* cortex, *ECX* entorhinal cortex, *RPKM* reads per kilobase of transcript per million mapped reads, *STG* superior temporal gyrus. *Red dashed line* denotes the Bonferroni significance, whilst *blue dashed line* denotes *p* < 0.05 in the *lower panel*

Having identified correlated mtDNA methylation patterns across different brain regions, we were interested to investigate whether we could identify differentially methylated regions (DMRs) in the mitochondrial genome between different regions of the cortex and cerebellum. To identify such tissue-specific DMRs within the mitochondrial genome, paired *t* tests were performed across matched cortical and cerebellum samples at 100 bp windows across the mitochondrial genome (see the Methods section). In total, we identified 74 nominally significant DMRs (*p* < 0.05) between the five individual cortical regions and the cerebellum (Table 2; Fig. 3). Of these DMRs, seven (Table 2, bold face) were found to be present across all prefrontal cortex areas (BA8, BA9, BA10). Furthermore, the direction of methylation difference was maintained in all Brodmann area regions, with three conserved regions of hypomethylation and four conserved regions of hypermethylation, with respect to the cerebellum. Furthermore, four of the seven conserved regions were adjacent to each other within the mitochondrial displacement loop (D-Loop) (16201–16600 bp), a region associated with gene transcription and DNA replication.

**Table 2:** List of DMRs identified between five anatomically discreet cortical regions and cerebellum

Start (bp)	Stop (bp)	Gene(s)	BA8		BA9		BA10		EC		STG	
			*p* value	Δ RPKM	*p* value	Δ RPKM	*p* value	Δ RPKM	*p* value	Δ RPKM	*p* value	Δ RPKM
1	100	D-Loop	**-**	-	**-**	-	**-**	**-**	**-**	**-**	**-**	**-**
101	200	D-Loop	**-**	-	**-**	-	**-**	**-**	**-**	**-**	**-**	**-**
201	300	D-Loop	**-**	-	**-**	-	**-**	**-**	**-**	**-**	**-**	**-**
301	400	D-Loop	**-**	-	**-**	-	**-**	**-**	**-**	**-**	**-**	**-**
401	500	D-Loop	**-**	-	**-**	-	**-**	**-**	**-**	**-**	**-**	**-**
501	600	MT-TF	**-**	-	**-**	-	**-**	**-**	**-**	**-**	**-**	**-**
601	700	MT-TF/MT-RNR1	**-**	-	**-**	-	**-**	**-**	**-**	**-**	**-**	**-**
701	800	MT-RNR1	-	-	-	-	-	-	3.21E−02	79156	-	**-**
801	900	MT-RNR1	4.31E−02	94246	2.93E−02	−43592	-	-	-	**-**	1.79E−02	95257
901	1000	MT-RNR1	ND	ND	ND	ND	ND	ND	ND	ND	ND	ND
1001	1100	MT-RNR1	ND	ND	ND	ND	ND	ND	ND	ND	ND	ND
1101	1200	MT-RNR1	ND	ND	ND	ND	ND	ND	ND	ND	ND	ND
1201	1300	MT-RNR1	ND	ND	ND	ND	ND	ND	ND	ND	ND	ND
1301	1400	MT-RNR1	ND	ND	ND	ND	ND	ND	ND	ND	ND	ND
1401	1500	MT-RNR1	ND	ND	ND	ND	ND	ND	ND	ND	ND	ND
1501	1600	MT-RNR1	ND	ND	ND	ND	ND	ND	ND	ND	ND	ND
1601	1700	MT-RNR1/MT-TV/MT-RNR2	ND	ND	ND	ND	ND	ND	ND	ND	ND	ND
1701	1800	MT-RNR2	ND	ND	ND	ND	ND	ND	ND	ND	ND	ND
1801	1900	MT-RNR2	ND	ND	ND	ND	ND	ND	ND	ND	ND	ND
1901	2000	MT-RNR2	ND	ND	ND	ND	ND	ND	ND	ND	ND	ND
2001	2100	MT-RNR2	ND	ND	ND	ND	ND	ND	ND	ND	ND	ND
2101	2200	MT-RNR2	ND	ND	ND	ND	ND	ND	ND	ND	ND	ND
2201	2300	MT-RNR2	ND	ND	ND	ND	ND	ND	ND	ND	ND	ND
**2301**	**2400**	**MT-RNR2**	**2.54E**−**02**	**−97351**	**3.20E**−**02**	**−29509**	**4.31E**−**02**	**−82048**	-	-	-	-
2401	2500	MT-RNR2	8.90E−03	70950	-	-	-	-	-	-	-	-
2501	2600	MT-RNR2	3.30E−03	51937	-	-	-	-	-	-	-	-
2601	2700	MT-RNR2	ND	ND	ND	ND	ND	ND	ND	ND	ND	ND
2701	2800	MT-RNR2	ND	ND	ND	ND	ND	ND	ND	ND	ND	ND
2801	2900	MT-RNR2	-	-	-	-	-	-	-	-	-	-
2901	3000	MT-RNR2	-	-	-	-	-	-	-	-	-	-
3001	3100	MT-RNR2	-	-	-	-	-	-	-	-	-	-
3101	3200	MT-RNR2	ND	ND	ND	ND	ND	ND	ND	ND	ND	ND
3201	3300	MT-RNR2/MT-TL	-	-	-	-	-	-	2.09E−02	242424	-	-
3301	3400	MT-TL1/MT-ND1	-	-	-	-	-	-	-	-	-	-
3401	3500	MT-ND1	-	-	1.54E−02	864685	-	-	-	-	-	-
3501	3600	MT-ND1	4.41E−02	1180914	-	-	1.72E−02	226066	-	-	-	-
**3601**	**3700**	**MT-ND1**	**4.73E**−**02**	**1250436**	**4.52E**−**02**	**1228936**	**3.73E**−**02**	**1183681**	-	-	-	-
3701	3800	MT-ND1	-	-	-	-	2.16E−02	20818	-	-	-	-
3801	3900	MT-ND1	-	-	-	-	-	-	-	-	-	-
3901	4000	MT-ND1	-	-	-	-	-	-	-	-	-	-
4001	4100	MT-ND1	2.41E−02	−39498	1.55E−02	−38704	-	-	4.68E−02	−49753	1.56E−02	243767
4101	4200	MT-ND1	ND	ND	ND	ND	ND	ND	ND	ND	ND	ND
4201	4300	MT-ND1/MT-TI	ND	ND	ND	ND	ND	ND	ND	ND	ND	ND
4301	4400	MT-TI/MT-TQ	ND	ND	ND	ND	ND	ND	ND	ND	ND	ND
4401	4500	MT-TM/MT-ND2	ND	ND	ND	ND	ND	ND	ND	ND	ND	ND
4501	4600	MT-ND2	ND	ND	ND	ND	ND	ND	ND	ND	ND	ND
4601	4700	MT-ND2	ND	ND	ND	ND	ND	ND	ND	ND	ND	ND
4701	4800	MT-ND2	ND	ND	ND	ND	ND	ND	ND	ND	ND	ND
4801	4900	MT-ND2	ND	ND	ND	ND	ND	ND	ND	ND	ND	ND
4901	5000	MT-ND2	ND	ND	ND	ND	ND	ND	ND	ND	ND	ND
5001	5100	MT-ND2	ND	ND	ND	ND	ND	ND	ND	ND	ND	ND
5101	5200	MT-ND2	ND	ND	ND	ND	ND	ND	ND	ND	ND	ND
5201	5300	MT-ND2	ND	ND	ND	ND	ND	ND	ND	ND	ND	ND
5301	5400	MT-ND2	ND	ND	ND	ND	ND	ND	ND	ND	ND	ND
5401	5500	MT-ND2	ND	ND	ND	ND	ND	ND	ND	ND	ND	ND
5501	5600	MT-ND2/MT-TW/MT-TA	ND	ND	ND	ND	ND	ND	ND	ND	ND	ND
5601	5700	MT-TA/MT-TN	ND	ND	ND	ND	ND	ND	ND	ND	ND	ND
5701	5800	MT-TN/MT-TC	ND	ND	ND	ND	ND	ND	ND	ND	ND	ND
5801	5900	MT-TC/MT-TY	ND	ND	ND	ND	ND	ND	ND	ND	ND	ND
5901	6000	MT-CO1	ND	ND	ND	ND	ND	ND	ND	ND	ND	ND
6001	6100	MT-CO1	ND	ND	ND	ND	ND	ND	ND	ND	ND	ND
6101	6200	MT-CO1	ND	ND	ND	ND	ND	ND	ND	ND	ND	ND
6201	6300	MT-CO1	ND	ND	ND	ND	ND	ND	ND	ND	ND	ND
6301	6400	MT-CO1	ND	ND	ND	ND	ND	ND	ND	ND	ND	ND
6401	6500	MT-CO1	ND	ND	ND	ND	ND	ND	ND	ND	ND	ND
6501	6600	MT-CO1	ND	ND	ND	ND	ND	ND	ND	ND	ND	ND
6601	6700	MT-CO1	ND	ND	ND	ND	ND	ND	ND	ND	ND	ND
6701	6800	MT-CO1	ND	ND	ND	ND	ND	ND	ND	ND	ND	ND
6801	6900	MT-CO1	ND	ND	ND	ND	ND	ND	ND	ND	ND	ND
6901	7000	MT-CO1	ND	ND	ND	ND	ND	ND	ND	ND	ND	ND
7001	7100	MT-CO1	ND	ND	ND	ND	ND	ND	ND	ND	ND	ND
7101	7200	MT-CO1	ND	ND	ND	ND	ND	ND	ND	ND	ND	ND
7201	7300	MT-CO1	ND	ND	ND	ND	ND	ND	ND	ND	ND	ND
7301	7400	MT-CO1	ND	ND	ND	ND	ND	ND	ND	ND	ND	ND
7401	7500	MT-CO1/MT-TS1	ND	ND	ND	ND	ND	ND	ND	ND	ND	ND
7501	7600	MT-TS1/MT-TD/MT-CO2	ND	ND	ND	ND	ND	ND	ND	ND	ND	ND
7601	7700	MT-CO2	ND	ND	ND	ND	ND	ND	ND	ND	ND	ND
7701	7800	MT-CO2	ND	ND	ND	ND	ND	ND	ND	ND	ND	ND
7801	7900	MT-CO2	ND	ND	ND	ND	ND	ND	ND	ND	ND	ND
7901	8000	MT-CO2	ND	ND	ND	ND	ND	ND	ND	ND	ND	ND
8001	8100	MT-CO2	ND	ND	ND	ND	ND	ND	ND	ND	ND	ND
8101	8200	MT-CO2	ND	ND	ND	ND	ND	ND	ND	ND	ND	ND
8201	8300	MT-CO2/MT-TK	ND	ND	ND	ND	ND	ND	ND	ND	ND	ND
8301	8400	MT-TK/MT-ATP8	ND	ND	ND	ND	ND	ND	ND	ND	ND	ND
8401	8500	MT-ATP8	ND	ND	ND	ND	ND	ND	ND	ND	ND	ND
8501	8600	MT-ATP8/MT-ATP6	ND	ND	ND	ND	ND	ND	ND	ND	ND	ND
8601	8700	MT-ATP6	ND	ND	ND	ND	ND	ND	ND	ND	ND	ND
8701	8800	MT-ATP6	ND	ND	ND	ND	ND	ND	ND	ND	ND	ND
8801	8900	MT-ATP6	ND	ND	ND	ND	ND	ND	ND	ND	ND	ND
8901	9000	MT-ATP6	ND	ND	ND	ND	ND	ND	ND	ND	ND	ND
9001	9100	MT-ATP6	ND	ND	ND	ND	ND	ND	ND	ND	ND	ND
9101	9200	MT-ATP6	ND	ND	ND	ND	ND	ND	ND	ND	ND	ND
9201	9300	MT-ATP6/MT-CO3	ND	ND	ND	ND	ND	ND	ND	ND	ND	ND
9301	9400	MT-CO3	ND	ND	ND	ND	ND	ND	ND	ND	ND	ND
9401	9500	MT-CO3	ND	ND	ND	ND	ND	ND	ND	ND	ND	ND
9501	9600	MT-CO3	ND	ND	ND	ND	ND	ND	ND	ND	ND	ND
9601	9700	MT-CO3	ND	ND	ND	ND	ND	ND	ND	ND	ND	ND
9701	9800	MT-CO3	ND	ND	ND	ND	ND	ND	ND	ND	ND	ND
9801	9900	MT-CO3	-	-	-	-	-	-	-	-	-	-
9901	10000	MT-CO3/MT-TG	-	-	-	-	-	-	-	-	-	-
10001	10100	MT-TG/MT-ND3	-	-	-	-	-	-	-	-	-	-
10101	10200	MT-ND3	-	-	-	-	-	-	-	-	-	-
10201	10300	MT-ND3	4.68E−02	−662126	-	-	-	-	3.14E−02	−591067	7.30E−03	−57012
**10301**	**10400**	**MT-ND3/MT-TR/MT-ND4L**	**3.61E**−**02**	**−132719**	**3.61E**−**02**	**−135759**	**4.02E**−**02**	**−115364**	1.40E−02	−73063	3.36E−02	−105106
10401	10500	MT-ND4L	-	-	4.68E−02	−71246	-	-	-	-	-	-
10501	10600	MT-ND4L	-	-	-	-	-	-	-	-	-	-
10601	10700	MT-ND4L	ND	ND	ND	ND	ND	ND	ND	ND	ND	ND
10701	10800	MT-ND4L/MT-ND4	ND	ND	ND	ND	ND	ND	ND	ND	ND	ND
10801	10900	MT-ND4	ND	ND	ND	ND	ND	ND	ND	ND	ND	ND
10901	11000	MT-ND4	ND	ND	ND	ND	ND	ND	ND	ND	ND	ND
11001	11100	MT-ND4	ND	ND	ND	ND	ND	ND	ND	ND	ND	ND
11101	11200	MT-ND4	ND	ND	ND	ND	ND	ND	ND	ND	ND	ND
11201	11300	MT-ND4	-	-	1.94E−02	−68903	1.84E−02	−79625	4.71E−02	58742	-	-
11301	11400	MT-ND4	-	-	4.78E−02	−139847	-	-	-	-	-	-
11401	11500	MT-ND4	ND	ND	ND	ND	ND	ND	ND	ND	ND	ND
11501	11600	MT-ND4	ND	ND	ND	ND	ND	ND	ND	ND	ND	ND
11601	11700	MT-ND4	ND	ND	ND	ND	ND	ND	ND	ND	ND	ND
11701	11800	MT-ND4	ND	ND	ND	ND	ND	ND	ND	ND	ND	ND
11801	11900	MT-ND4	ND	ND	ND	ND	ND	ND	ND	ND	ND	ND
11901	12000	MT-ND4	ND	ND	ND	ND	ND	ND	ND	ND	ND	ND
12001	12100	MT-ND4	ND	ND	ND	ND	ND	ND	ND	ND	ND	ND
12101	12200	MT-ND4/MT-TH	ND	ND	ND	ND	ND	ND	ND	ND	ND	ND
12201	12300	MT-TS2/MT-TL2	ND	ND	ND	ND	ND	ND	ND	ND	ND	ND
12301	12400	MT-TL2/MT-ND5	4.71E−02	1787	-	−197592	-	-	-	-	-	-
12401	12500	MT-ND5	-	-	-	-	-	-	-	-	-	-
12501	12600	MT-ND5	-	-	-	-	-	-	-	-	-	-
12601	12700	MT-ND5	-	-	-	-	-	-	-	-	-	-
12701	12800	MT-ND5	2.05E−02	−5702	-	-	4.58E−02	−388706	-	-	4.36E−02	−394978
12801	12900	MT-ND5	2.26E−02	−89668	-	-	-	-	-	-	-	-
12901	13000	MT-ND5	-	-	3.10E−02	−130016	-	-	-	-	-	-
13001	13100	MT-ND5	ND	ND	ND	ND	ND	ND	ND	ND	ND	ND
13101	13200	MT-ND5	ND	ND	ND	ND	ND	ND	ND	ND	ND	ND
13201	13300	MT-ND5	ND	ND	ND	ND	ND	ND	ND	ND	ND	ND
13301	13400	MT-ND5	1.48E−02	−175917	-	-	9.80E−03	−144949	3.40E−02	−133694	-	-
13401	13500	MT-ND5	3.64E−02	−104010	-	-	3.40E−02	−81264	-	-	-	-
13501	13600	MT-ND5	-	-	-	-	-	-	-	-	-	-
13601	13700	MT-ND5	-	-	-	-	-	-	-	-	-	-
13701	13800	MT-ND5	1.31E−02	−422610	-	-	1.20E−03	3714	-	-	-	-
13801	13900	MT-ND5	-	-	3.72E−02	708761	2.59E−02	123249	-	-	-	-
13901	14000	MT-ND5	-	-	-	-	2.17E−02	−75118	-	-	-	-
14001	14100	MT-ND5	-	-	-	-	-	-	-	-	-	-
14101	14200	MT-ND5/MT-ND6	-	-	-	-	-	-	-	-	4.18E−02	−534766
14201	14300	MT-ND6	-	-	-	-	-	-	-	-	-	-
14301	14400	MT-ND6	-	-	-	-	-	-	-	-	-	-
14401	14500	MT-ND6	-	-	-	-	-	-	-	-	-	-
14501	14600	MT-ND6	-	-	-	-	-	-	-	-	4.86E−02	−82767
14601	14700	MT-ND6/MT-TE	-	-	-	-	-	-	-	-	-	-
14701	14800	MT-TE/MT-CYB	-	-	-	-	-	-	-	-	-	-
14801	14900	MT-CYB	ND	ND	ND	ND	ND	ND	ND	ND	ND	ND
14901	15000	MT-CYB	ND	ND	ND	ND	ND	ND	ND	ND	ND	ND
15001	15100	MT-CYB	ND	ND	ND	ND	ND	ND	ND	ND	ND	ND
15101	15200	MT-CYB	-	-	-	-	-	-	-	-	-	-
15201	15300	MT-CYB	-	-	-	-	-	-	1.20E−02	254183	-	-
15301	15400	MT-CYB	-	-	-	-	-	-	-	-	-	-
15401	15500	MT-CYB	4.38E−02	10628	-	-	2.10E−02	−554527	-	-	3.28E−02	−554225
15501	15600	MT-CYB	3.73E−02	−671707	-	-	1.23E−02	−685630	3.82E−02	9301	3.51E−02	−695515
15601	15700	MT-CYB	-	-	-	-	4.33E−02	−746270	-	-	-	-
15701	15800	MT-CYB	-	-	-	-	-	-	-	-	-	-
15801	15900	MT-CYB/MT-TT	-	-	-	-	-	-	-	-	-	-
15901	16000	MT-TT/MT-TP	-	-	-	-	-	-	-	-	-	-
16001	16100	MT-TP	-	-	-	-	-	-	-	-	-	-
16101	16200	D-Loop	-	-	3.40E−03	3014	-	-	-	-	-	-
**16201**	**16300**	**D-Loop**	**4.64E**−**02**	**380407**	**2.50E**−**02**	**1117943**	**3.68E**−**02**	**296890**	-	-	-	-
**16301**	**16400**	**D-Loop**	**3.65E**−**02**	**−941720**	**1.38E**−**02**	**−112453**	**2.73E**−**02**	**−177146**	-	-	-	-
**16401**	**16500**	**D-Loop**	**4.29E**−**02**	**444051**	**2.20E**−**02**	**1151402**	**1.68E**−**02**	**313156**	-	-	-	-
**16501**	**16600**	**D-Loop**	**3.71E**−**02**	**784572**	**2.10E**−**02**	**764994**	**2.46E**−**02**	**836662**	-	-	-	-

Shown is the location of the DMR within the mitochondrial genome (ChrM) (based on GENCODE), the gene(s) residing within the 100 bp window, and *p* value from paired *t* tests between each of the five cortical regions: Brodmann areas 8, 9 and 10 (BA8, BA9, BA10), entorhinal cortex (ECX) and superior temporal gyrus (STG) compared to the cerebellum (CER). Results are displayed in order of genomic position. RPKM and corresponding *p* values are shown for windows if *p* < 0.05. Key: - denotes data not significant (*p* > 0.05); ND denotes not determined as the window was not included in analysis due to removal in *NUMT* pipeline. Results shown in bold represent those found to be present across all prefrontal cortex areas (BA8, BA9, BA10)

### A number of differentially methylated regions in mtDNA can be observed between the cortex and cerebellum

**Fig. 4:**
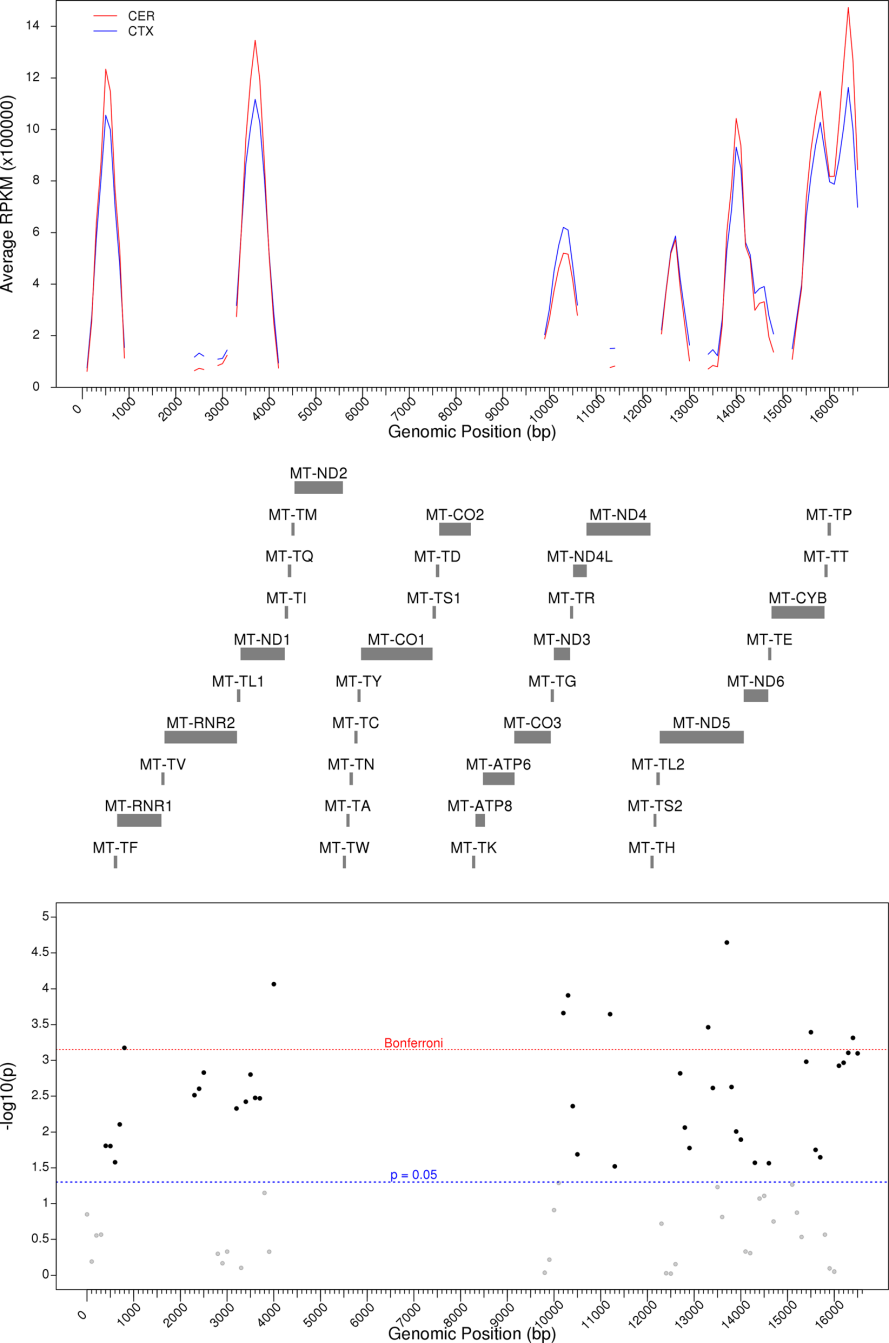
DNA methylation differences are seen in the mitochondrial genome between the cerebellum and cortex. RPKM values in the total cortex and cerebellum across the mitochondrial genome are shown in the *top panel*, with gene positions downloaded from GENCODE shown in the *middle panel*. For each 100 bp window, paired *t* tests were performed to compare the cortex to the cerebellum, with -log10 (*p*) shown in the *bottom panel. BLD* blood, *BA8* Brodmann area 8, *BA9* Brodmann area 9, *BA10* Brodmann area 10, *CER* cerebellum, *CTX* cortex, *ECX* entorhinal cortex, *RPKM* reads per kilobase of transcript per million mapped reads, *STG* superior temporal gyrus. *Red dashed line* denotes the Bonferroni significance, whilst *blue dashed line* denotes *p* < 0.05 in the *lower panel*

We were also interested to see whether total cortical tissue was significantly different to matched cerebellum samples. Given the paired nature of the different anatomical regions of the cortex, we used a multilevel mixed effects model to compare total cortex to cerebellum (see the Methods section). This analysis revealed 48 nominally significant (*p* < 0.05) windows (Table 3; Fig. 4), of which eight passed the Bonferroni correction (Table 3, bold face). Interestingly, three of these eight were adjacent to each other, lying between 10301 and 10600 bp and covering MT-ND3/MT-ND4L and MT-TR. We also saw a Bonferroni significant difference in DNA methylation in the D-Loop, where we earlier noted DNA methylation changes across all three Brodmann area regions.

**Table 3:** List of DMRs identified between total cortex and cerebellum

Start (bp)	Stop (bp)	Gene(s)	*p* value
1	100	D-Loop	-
101	200	D-Loop	-
201	300	D-Loop	-
301	400	D-Loop	-
401	500	D-Loop	7.99E−03
501	600	MT-TF	4.49E−03
601	700	MT-TF/MT-RNR1	1.16E−02
701	800	MT-RNR1	3.22E−03
**801**	**900**	**MT-RNR1**	**1.91E**−**04**
901	1000	MT-RNR1	ND
1001	1100	MT-RNR1	ND
1101	1200	MT-RNR1	ND
1201	1300	MT-RNR1	ND
1301	1400	MT-RNR1	ND
1401	1500	MT-RNR1	ND
1501	1600	MT-RNR1	ND
1601	1700	MT-RNR1/MT-TV/MT-RNR2	ND
1701	1800	MT-RNR2	ND
1801	1900	MT-RNR2	ND
1901	2000	MT-RNR2	ND
2001	2100	MT-RNR2	ND
2101	2200	MT-RNR2	ND
2201	2300	MT-RNR2	ND
2301	2400	MT-RNR2	8.10E−03
2401	2500	MT-RNR2	7.50E−03
2501	2600	MT-RNR2	5.14E−03
2601	2700	MT-RNR2	ND
2701	2800	MT-RNR2	ND
2801	2900	MT-RNR2	-
2901	3000	MT-RNR2	-
3001	3100	MT-RNR2	-
3101	3200	MT-RNR2	-
3201	3300	MT-RNR2/MT-TL	1.51E−03
3301	3400	MT-TL1/MT-ND1	-
3401	3500	MT-ND1	1.37E−02
3501	3600	MT-ND1	4.27E−03
3601	3700	MT-ND1	5.56E−03
3701	3800	MT-ND1	8.21E−03
3801	3900	MT-ND1	-
3901	4000	MT-ND1	-
**4001**	**4100**	**MT-ND1**	**3.07E**−**06**
4101	4200	MT-ND1	-
4201	4300	MT-ND1/MT-TI	ND
4301	4400	MT-TI/MT-TQ	ND
4401	4500	MT-TM/MT-ND2	ND
4501	4600	MT-ND2	ND
4601	4700	MT-ND2	ND
4701	4800	MT-ND2	ND
4801	4900	MT-ND2	ND
4901	5000	MT-ND2	ND
5001	5100	MT-ND2	ND
5101	5200	MT-ND2	ND
5201	5300	MT-ND2	ND
5301	5400	MT-ND2	ND
5401	5500	MT-ND2	ND
5501	5600	MT-ND2/MT-TW/MT-TA	ND
5601	5700	MT-TA/MT-TN	ND
5701	5800	MT-TN/MT-TC	ND
5801	5900	MT-TC/MT-TY	ND
5901	6000	MT-CO1	ND
6001	6100	MT-CO1	ND
6101	6200	MT-CO1	ND
6201	6300	MT-CO1	ND
6301	6400	MT-CO1	ND
6401	6500	MT-CO1	ND
6501	6600	MT-CO1	ND
6601	6700	MT-CO1	ND
6701	6800	MT-CO1	ND
6801	6900	MT-CO1	ND
6901	7000	MT-CO1	ND
7001	7100	MT-CO1	ND
7101	7200	MT-CO1	ND
7201	7300	MT-CO1	ND
7301	7400	MT-CO1	ND
7401	7500	MT-CO1/MT-TS1	ND
7501	7600	MT-TS1/MT-TD/MT-CO2	ND
7601	7700	MT-CO2	ND
7701	7800	MT-CO2	ND
7801	7900	MT-CO2	ND
7901	8000	MT-CO2	ND
8001	8100	MT-CO2	ND
8101	8200	MT-CO2	ND
8201	8300	MT-CO2/MT-TK	ND
8301	8400	MT-TK/MT-ATP8	ND
8401	8500	MT-ATP8	ND
8501	8600	MT-ATP8/MT-ATP6	ND
8601	8700	MT-ATP6	ND
8701	8800	MT-ATP6	ND
8801	8900	MT-ATP6	ND
8901	9000	MT-ATP6	ND
9001	9100	MT-ATP6	ND
9101	9200	MT-ATP6	ND
9201	9300	MT-ATP6/MT-CO3	ND
9301	9400	MT-CO3	ND
9401	9500	MT-CO3	ND
9501	9600	MT-CO3	ND
9601	9700	MT-CO3	ND
9701	9800	MT-CO3	ND
9801	9900	MT-CO3	-
9901	10000	MT-CO3/MT-TG	-
10001	10100	MT-TG/MT-ND3	1.81E−02
10101	10200	MT-ND3	1.39E−02
**10201**	**10300**	**MT-ND3**	**3.53E**−**04**
**10301**	**10400**	**MT-ND3/MT-TR/MT-ND4L**	**1.19E**−**05**
**10401**	**10500**	**MT-ND4L**	**2.61E**−**04**
10501	10600	MT-ND4L	9.05E−04
10601	10700	MT-ND4L	ND
10701	10800	MT-ND4L/MT-ND4	ND
10801	10900	MT-ND4	ND
10901	11000	MT-ND4	ND
11001	11100	MT-ND4	ND
11101	11200	MT-ND4	ND
**11201**	**11300**	**MT-ND4**	**8.86E**−**05**
11301	11400	MT-ND4	1.65E−03
11401	11500	MT-ND4	ND
11501	11600	MT-ND4	ND
11601	11700	MT-ND4	ND
11701	11800	MT-ND4	ND
11801	11900	MT-ND4	ND
11901	12000	MT-ND4	ND
12001	12100	MT-ND4	ND
12101	12200	MT-ND4/MT-TH	ND
12201	12300	MT-TS2/MT-TL2	ND
12301	12400	MT-TL2/MT-ND5	-
12401	12500	MT-ND5	-
12501	12600	MT-ND5	-
12601	12700	MT-ND5	-
12701	12800	MT-ND5	1.81E−03
12801	12900	MT-ND5	1.05E−02
12901	13000	MT-ND5	8.23E−03
13001	13100	MT-ND5	ND
13101	13200	MT-ND5	ND
13201	13300	MT-ND5	ND
13301	13400	MT-ND5	1.44E−03
13401	13500	MT-ND5	9.13E−04
13501	13600	MT-ND5	1.61E−02
13601	13700	MT-ND5	3.89E−02
**13701**	**13800**	**MT-ND5**	**2.77E**−**04**
13801	13900	MT-ND5	2.80E−03
13901	14000	MT-ND5	1.88E−02
14001	14100	MT-ND5	9.31E−03
14101	14200	MT-ND5/MT-ND6	-
14201	14300	MT-ND6	-
14301	14400	MT-ND6	1.04E−02
14401	14500	MT-ND6	1.99E−02
14501	14600	MT-ND6	2.26E−02
14601	14700	MT-ND6/MT-TE	3.82E−03
14701	14800	MT-TE/MT-CYB	1.92E−02
14801	14900	MT-CYB	ND
14901	15000	MT-CYB	ND
15001	15100	MT-CYB	ND
15101	15200	MT-CYB	3.30E−02
15201	15300	MT-CYB	-
15301	15400	MT-CYB	-
15401	15500	MT-CYB	8.52E−04
15501	15600	MT-CYB	7.43E−04
15601	15700	MT-CYB	1.16E−02
15701	15800	MT-CYB	2.24E−02
15801	15900	MT-CYB/MT-TT	-
15901	16000	MT-TT/MT-TP	-
16001	16100	MT-TP	-
16101	16200	D-Loop	2.23E−03
**16201**	**16300**	**D-Loop**	**5.02E**−**04**
16301	16400	D-Loop	1.84E−03
16401	16500	D-Loop	1.12E−03
16501	16600	D-Loop	2.20E−03

Shown is the location of the DMR within ChrM (based on GENCODE), the gene(s) residing within the 100 bp window, and *p* value from a multilevel mixed effects model. Results are displayed in order of genomic position. RPKM and corresponding *p* values are shown for windows if *p* < 0.05. Key: - denotes data not significant (*p* > 0.05); ND denotes not determined as the window was not included in analysis due to removal in *NUMT* pipeline; bold denotes windows that reached our Bonferroni significant threshold of *p* < 7.04E−04

### MtDNA methylation patterns can distinguish between tissue types

**Fig. 5:**
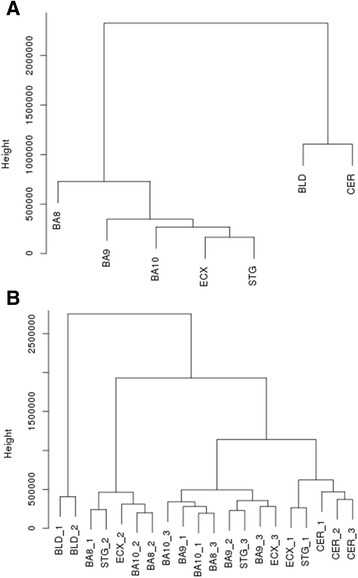
MtDNA methylation patterns can distinguish between tissue types. **a** Average RPKM values for each cortical brain region, cerebellum and blood samples were clustered based upon the Euclidean distance, identifying two major clusters; the cortex and blood-cerebellum. **b** When clustering RPKM values in the individual samples from the cortex, cerebellum and blood, we observed that individual cortex samples clustered together, whilst cerebellum and blood samples formed separate clusters. This highlights that tissue-specific differences between the cortex, cerebellum and blood are greater than intra-individual variability within a tissue. *BLD* blood, *BA8* Brodmann area 8, *BA9* Brodmann area 9, *BA10* Brodmann area 10, *CER* cerebellum, *CTX* cortex, *ECX* entorhinal cortex, *RPKM* reads per kilobase of transcript per million mapped reads, *STG* superior temporal gyrus

Although we have shown that mtDNA methylation patterns are highly similar between distinct anatomical regions of the human brain and blood, we were also interested to identify whether mtDNA methylation patterns could distinguish between these tissue types. Through unsupervised hierarchical clustering, we showed that average mtDNA methylation patterns can segregate these tissues (Fig. 5a). Importantly, ncDNA methylation profiles in the same samples have also been previously shown to separate the cortex, cerebellum and blood [35]. Interestingly, when we performed unsupervised hierarchical clustering on the individual samples, we found that, in most cases, intra-individual differences across tissue types are greater than inter-individual differences within each tissue type, as the cortex, cerebellum and blood samples clustered with their own tissue type, respectively (Fig. 5b).

## Discussion

The availability of publicly available epigenomic data provides a great resource for mitochondrial epigenetics, a field that is relatively nascent and has yet to be thoroughly investigated in a range of complex diseases. Here, we present evidence that mtDNA methylation patterns across mtDNA are brain region specific. However, data such as that presented here is confounded by a lack of isolation of mtDNA prior to antibody enrichment and sequencing. As such, the potential of including *NUMT*s in datasets derived from data generated using total genomic DNA could lead to misleading results. Here, we controlled for regions of high sequence homology between the nuclear and mitochondrial genomes. However, this approach is likely over-conservative and does lead to the generation of a somewhat truncated consensus sequence. PCA of the mitochondrial epigenome after corrections for nuclear homology was able to separate individuals belonging to the three main tissue types, the blood, cortex and cerebellum based on mtDNA methylation variation among tissue types. This tissue specificity is further highlighted by the identification of eight DMRs that pass the Bonferroni correction for multiple testing between total cortex and cerebellum. MtDNA methylation has been shown to be cell line dependent in the past. [31] Although overall DNA methylation levels were low in all tissues, it is worth noting that the study was performed on non bisulfite-treated DNA. As such, the low percentage of mtDNA methylation is not a pitfall due to a lack of a total bisulfite treatment efficiency. One limitation of the current study is the unavailability of publicly available MeDIP-seq datasets of matched cortical and cerebellum tissue from other cohorts for validation purposes. Future work would aim to replicate our findings in additional study cohorts and also to investigate the relationship between mitochondrial DNA methylation and gene expression.

Despite a number of nominally significant windows being identified between each individual cortical region and the cerebellum, these did not pass the Bonferroni correction, although it is likely this method is too stringent. Nevertheless, the conservation of seven nominally significant windows across each Brodmann area is interesting to note. Four of these windows lie adjacent to each other and correspond to the mitochondrial D-Loop, a region containing the only two mitochondrial promoters which is typically associated with gene transcription and DNA replication. However, one limitation of this study is owed to the use of antibody-based enrichment, resulting in the analysis being limited to a window-based approach. Despite this, studies of the nuclear genome have shown high correlation between window-based approaches and, more sensitive, single-site assays such as the Illumina 450K beadarray [32]. However, given the small size of the mitochondrial genome and that 23 of the 37 genes present in the genome are below 100 bp in size, this window-based approach may not be the most appropriate for future studies designed to specifically assess mtDNA methylation as it can result in a window intersecting two genes in the polycistronic transcript.

## Conclusions

This method provides a conservative approach to determine mtDNA methylation across the genome for data previously generated using next-generation sequencing approaches such as MeDIP-seq. Its conservative nature reduces the risk of the inclusion of *NUMT*s in the final analysis of whole genome data but may also lead to the inclusion of false negatives as well as potential gaps in the reference sequence. As such, it is best suited to analyzing previously generated whole genome data and is not a replacement for the isolation of mitochondrial DNA [36] prior to targeted methylation studies, which would be the optimal approach for investigating mitochondrial epigenomics. However, our method has allowed the identification of novel brain-region-specific DMRs in a previously generated publicly available dataset. Furthermore, the identification of brain region-specific mtDNA methylation patterns across the mitochondrial epigenome suggests the importance of a focussed, tissue-specific study design when investigating mtDNA methylation. As previously discussed, one caveat when utilizing MeDIP-seq data is the segregation of data into neighbouring windows, meaning that determining the exact corresponding gene of a DMR is difficult and, as such, future studies should aim to sequence the mitochondrial DNA methylome at single-base resolution to address this.

## Methods

### Data collection

We utilized publicly available MeDIP-seq data from Davies et al. [35]. In brief, this data was generated using 5 μg fragmented gDNA, which, following end repair <A> base addition and adaptor ligation, was immunoprecipitated using an anti-5-mC antibody (Diagenode, Liège, Belgium). MeDIP DNA was purified and then amplified using adaptor-mediated PCR, with DNA fragments between 220 and 320 bp subjected to highly parallel 50 bp paired-end sequencing on the Illumina Hi-Seq platform. The paired-end, raw fasta files were provided by the authors and quality checked using FastQC. Sample information is provided in Table 1.

### Quality control and *NUMT* exclusion

Fasta files were subjected to adaptor and Phred score (*q* < 20) trimming. In an attempt to remove any potential contamination of possible *NUMT*s, multiple alignments to the reference genome were undertaken. Paired fasta files were aligned to GRCH37 using BWA. Unique and mapped reads aligning to the mitochondria were then re-mapped to a custom GRCH37 reference without the mitochondrial chromosome. Reads not mapping to the custom reference were then taken forward and realigned to the full GRCH37 reference to eliminate the possibility of homologous regions mapping falsely to the mitochondrial genome (Fig. 1). All alignments were carried out using BWA mem and default settings. Reads per kilobase of transcript per million mapped reads (RPKM) values for each sample were calculated using the MEDIPS package [37]. Methylation was averaged across 100 bp non-overlapping windows (default parameter setting in MEDIPS), and only windows with read counts >10 were considered for analysis. Due to the non-normal distribution of all cohorts, RPKM values were log^2^ transformed before statistical analysis.

### Statistical analyses

All analyses were performed in the *R* statistical environment version 3.2.1 [38]. For all analyses, a nominally significant threshold of *p* < 0.05 and a Bonferroni significant threshold of *p* < 7.04E−04 were used. Given the matched sample nature of this cohort, two-tailed, paired *t* tests were performed at each window along the mitochondrial genome to identify DMRs between the individual cortical regions and cerebellum. To compare the total cortex to cerebellum, we performed a multilevel mixed effects model in the Lme4 package in *R* [39], using the brain region as the random effect and individual as the fixed effect. To assess the similarity of the brain regions, we used the *R* function “hclust” to cluster average RPKM values for the brain regions using the Euclidean distance. We used the *R* function “corrgram” within the corrgram package [40] to order samples based upon the similarity of their principal components.

## Abbreviations

5-mC: 5-methylcytosine
BA: Brodmann area
DMR: Differentially methylated region
MeDIP-seq: Methylated DNA Immunoprecipitation Sequencing
mtDNA: Mitochondrial DNA
ncDNA: Nuclear DNA
*NUMT*s: Nuclear-mitochondrial DNA
PCA: Principal component analysis
rRNA: Ribosomal ribonucleic acid
tRNA: Transfer ribonucleic acid
